# Adipocytes regulate monocyte development through the OGT-NEFA-CD36/FABP4 pathway in high-fat diet-induced obesity

**DOI:** 10.1038/s41419-025-07721-x

**Published:** 2025-05-19

**Authors:** Na He, Yingjie Li, Fabao Liu, Xifeng Dong, Daoxin Ma

**Affiliations:** 1 Advanced Medical Research Institute, Shandong University, Shandong, 250012 China; 2 Department of Hematology, Qilu Hospital of Shandong University, Shandong, 250012 China; 3 Department of Health Management Center, Qilu Hospital of Shandong University, Shandong, 250012 China; 4 Department of Hematology, Tianjin Medical University General Hospital, Tianjin, 300052 China

**Keywords:** Cell biology, Haematopoiesis

## Abstract

Obesity, resulting from excessive adipocyte accumulation, is a primary risk for various diseases. Although its impact on hematopoietic stem cell (HSC) function has been reported, its effects on HSC differentiation remain controversial. O-GlcNAc transferase (OGT), which catalyzes the attachment of N-acetylglucosamine to serine and threonine residues in proteins, acts as a metabolic sensor capable of regulating diverse physiological processes. This study demonstrates that obesity is associated with higher peripheral monocyte levels. Adipocyte OGT is crucial for monocyte development in high-fat diet (HFD)-induced obesity, promoting an increase in peripheral blood monocytes through transcriptional activation of nonesterified fatty acids (NEFA), a critical energy substrate. Loss of adipocyte OGT decreases serum NEFA levels, reduces white adipose tissue, and inhibits HSC differentiation into monocytes in HFD-induced obesity. Mechanistically, the regulated effect of adipocyte OGT on monocyte development may be mediated by NEFA-cluster of differentiation 36/fatty acid binding protein 4 (CD36/FABP4) pathway in HSCs in HFD-induced obesity. These findings establish the critical role of adipocyte OGT in hematopoietic homeostasis and monocyte development.

## Introduction

Obesity has become a significant global public health issue. The prevalence of excessive weight gain has doubled globally, and approximately one-third of the world’s population is now classified as obese or overweight [1]. Obesity not only increases susceptibility to a variety of diseases but also affects the cell composition in the bone marrow (BM), seriously damages the function of hematopoietic stem cells (HSCs). Obesity may be associated with an increased risk of developing leukemia [2] and multiple myeloma [3].

HSCs are considered the most crucial component of the hematopoietic system. They are at the top of the hematopoietic hierarchy, with long term self-renewal ability and multipotent differentiation potential [4]. It is long and widely believed that adipocytes inhibit HSCs differentiation [5]. In high-fat diet (HFD)-fed db/db mice, the density of BM cells significantly decreased with the accumulation of BM adipocytes (BMAds) [6]. Although the excessive accumulation of BMAds is generally considered detrimental to hematopoietic function, some research proposes that adipocytes play an active role in hematopoietic homeostasis. It has been reported that lymphocytes were increased by 10–18% and positively correlated to BM adipocytes in obese mice, which may be due to the higher levels of leptin secreted by adipocytes [7]. Other studies also support the idea that obesity, induced by a HFD, leads to an increase in the number of HSCs that differentiate into BM precursor cells [8]. Therefore, the role of obesity in HSCs differentiation remains controversial, and the specific molecular mechanisms are still not well understood.

O-GlcNAc transferase (OGT) is the only enzyme in the human genome that modifies target protein serine and threonine residues with a single O-GlcNAc sugar. Metabolic homeostasis is intricately linked to O-GlcNAc homeostasis. The hexosamine biosynthesis pathway (HBP) supplies UDP-GlcNAc as a substrate to OGT [9, 10]. It has been reported that adipocyte OGT reactivates lipid desaturation, leading to increased accumulation of endocannabinoids in adipose tissue [11]. However, it remains unknown whether OGT in adipocytes influences HSC differentiation, particularly in an obesity-prone environment. In this study, we demonstrate that adipocyte OGT activation prompts nonesterified fatty acids (NEFA) secretion and enhances the differentiation of HSCs into monocytes via an adipose-to-NEFA signaling axis during HFD feeding. These findings suggest that adipocyte OGT acts as a fat sensor, activating the hematopoietic system and promoting the differentiation and maturation of monocytes.

## Results

### Obesity as an independent factor influencing peripheral monocyte levels

**Fig. 1:**
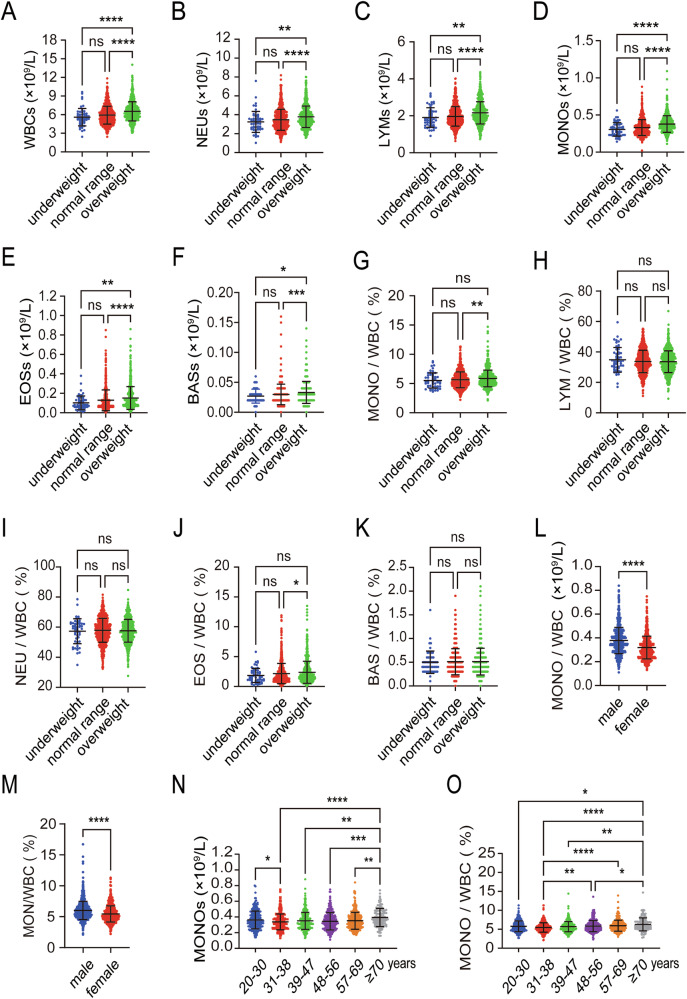
Obesity as an independent factor influencing peripheral monocyte levels. **A**–**F** Absolute values of WBC, NEU, LYM, MONO, EOS, and BAS in the underweight, normal range, and overweight groups in peripheral blood. **G**–**K** Proportions of MONO/WBC, LYM/WBC, NEU/WBC, EOS/WBC, and BAS/WBC in the underweight, normal range, and overweight groups in peripheral blood. **L** Absolute values of MONO in male and female groups in peripheral blood. **M** Proportions of MONO/WBC in male and female groups in peripheral blood. **N** Absolute values of MONO across different age groups in peripheral blood. **O** Proportions of MONO/WBC across different age groups in peripheral blood. Data are presented as mean ± SEM. *p < 0.05, **p < 0.01, ***p < 0.001, ****p < 0.0001, ns not significant.

Obesity has been reported to influence hematogenesis, but its detailed effect on the blood differentiation remains controversial. We first analyzed the complete blood count (CBC) of 1671 adult subjects and correlated it with obesity. Compared with the underweight or normal weight group, a significant increase was found in the overweight group for the absolute values of several key blood indicators, including white blood cells (WBC), red blood cells (RBC), blood platelets (PLT), neutrophils (NEU), monocytes (MONO), lymphocytes (LYM), eosinophils (EOS), and basophils (BAS) (Fig. 1A–F and Supplementary Fig. 1A, B). Interestingly, as for the proportion of blood cells, only MONO within the WBC count (MONO/WBC) significantly increased in the overweight group, whereas other WBC subsets showed no significant change (Fig. 1G–K). These results indicate that obesity is associated with increased peripheral monocytes.

Moreover, other factors such as gender, age, hormone levels, aging, and immunity have been shown to influence the variations of CBC or body mass index (BMI) [12]. Our findings revealed that the BMI of male was significantly higher than that of female, and both the absolute value and proportion of peripheral monocytes were higher in the male compared to the female (Fig. 1L, M and Supplementary Fig. 1C). Subsequently, we correlated BMI with age and found that middle-aged and elderly individuals had higher BMI compared to younger individuals. Similarly, both the absolute value and proportion of peripheral monocytes increased with age (Fig. 1N, O and Supplementary Fig. 1D). These results suggest that gender and age significantly influence the increase of monocytes in peripheral blood. Therefore, to determine whether BMI is independently associated with monocyte formation, a multivariate linear regression analysis was conducted. After adjusting for age and gender, we found that BMI positively correlated with peripheral WBC count and monocyte count, indicating that BMI might relate to WBC and monocyte formation (Table 1). Together, these observations suggest that obesity might contribute to the differentiation of peripheral monocytes and induce the increase of monocytes.

**Table 1:** Linear regression analysis adjusted for age, gender, and BMI.

Independent variable	White blood cell count (10^9^/L) β (95% CI), *p*	Monocyte cell count (10^9^/L) β (95% CI), *p*
Gender	−0.537 (−0.709 — −0.366), <0.001	−0.055 (−0.066 — −0.044), <0.001
Age	−0.473 (0.273 — −0.674), <0.001	0.029 (0.014 — −0.044), <0.001
BMI	0.03 (0.014 — 0.047), <0.001	0.004 (0.002 — 0.005), <0.001

*CI* Confidence Interval, *BMI* body mass index.

### HFD-induced obesity promotes the increase of monocytes in vivo, and correlates with peripheral dyslipidemia

**Fig. 2:**
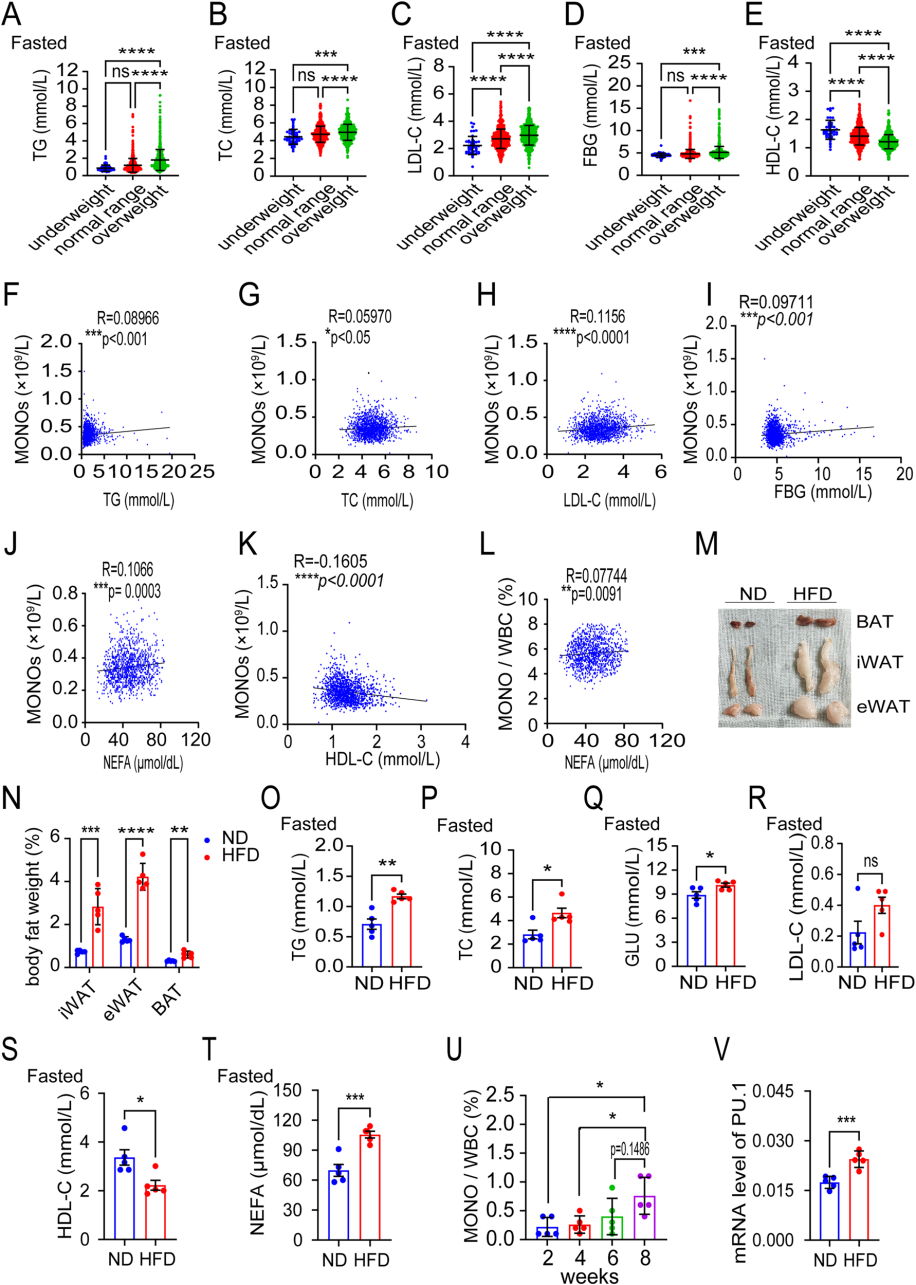
HFD-induced obesity promotes the increase of monocytes in vivo, and correlates with peripheral dyslipidemia. **A**–**E** Serum levels of TG, TC, LDL-C, FBG and HDL-C in the underweight, normal weight, and overweight groups in 12 h fasted state. **F**–**K** Correlation analysis of serum levels of TG, TC, LDL-C, FBG, NEFA and HDL-C with absolute values of MONO in peripheral blood. **L** Correlation analysis of serum levels of NEFA with proportions of MONO/WBC in peripheral blood. **M** Representative images of BAT, iWAT, and eWAT in the ND (*n* = 5) and HFD (*n* = 5) groups. **N** Tissue weights, presented as % of body weight in mice described in (**M**) (ND group *n* = 5; HFD group *n* = 5). **O**–**T** Serum levels of TG, TC, GLU, LDL-C, HDL-C and NEFA in the ND (*n* = 5) and HFD (*n* = 5) groups in 12 h fasted state. **U** The change in the proportion of monocytes to WBCs in PB of mice in the HFD (*n* = 5) group during HFD feeding for 8 weeks. **V** mRNA levels of PU.1 in HSCs from ND (*n* = 5) and HFD groups (*n* = 5). Data are presented as mean ± SEM. *p < 0.05, **p < 0.01, ***p < 0.001, ****p < 0.0001, ns not significant.

Although the association between obesity and metabolic complications is well-documented [13, 14], the specific impact of obesity on peripheral lipid metabolism remains under debate. By analyzing a large sample of data within 1671 adult subjects, we found that serum level of triglycerides (TG), total cholesterol (TC), low-density lipoprotein cholesterol (LDL-C), or fasting blood glucose (FBG) in the overweight group were significantly higher than that in the underweight and normal weight groups (Fig. 2A–D). Moreover, these indicators are positively correlated with the level of BMI (Supplemental Fig. 2A–D). Meanwhile, we found that the serum level of high-density lipoprotein cholesterol (HDL-C) was decreased and negatively correlated with BMI (Fig. 2E and Supplementary Fig. 2E). These data suggest that obesity might contribute to the peripheral dyslipidemia. In addition, we found that the absolute number of peripheral monocytes was positively correlated with TG, TC, LDL-C, FBG and NEFA (Fig. 2F–J), and negatively correlated with HDL-C (Fig. 2K). Interestingly, the proportion of peripheral monocytes was positively correlated with TG and NEFA (Fig. 2L and Supplementary Fig. 2F), but not significantly correlated with other indicators (Supplementary Fig. 2G–J). Together, obesity-associated serum dyslipidemia, especially TG and NEFA, might contribute to the increase of peripheral monocytes.

To further elucidate the mechanisms underlying obesity-mediated monocyte increase, we applied high-fat diet (HFD) for 8 weeks to establish a murine obesity model while used mice fed with chow-fed (normal diet, ND) as controls. Our results showed that mouse body weight increased significantly in the HFD group compared with controls during the feeding period (Supplementary Fig. 2K). Subsequently, three kinds of murine adipose tissues including inguinal white adipose tissue (iWAT), epididymal white adipose tissue (eWAT), and brown adipose tissue (BAT), were harvested (Fig. 2M). As they represent very different aspects of obesity and energy homeostasis, we separately weighted and analyzed the percentage of the three adipose tissues to body weight, which related to the collective mass of the three fat depots dissected but not represented whole body fat mass. Our results showed that any of the three adipose tissue percentages in HFD mice was significantly higher than that in the ND mice (Fig. 2N). Blood biochemistry was analyzed in both ND and HFD groups. Consistent with the human results, the levels of TG, TC, LDL-C and FBG were significantly increased in HFD-induced obesity mice compared with those in ND mice (Fig. 2O–R), while HDL-C was dramatically reduced (Fig. 2S). Obesity, especially obesity-induced insulin resistance, has been reported to be responsible for the increased peripheral blood FFAs released from adipose tissue [13, 15], so we further determined the level of NEFA. Consequently, NEFA was found significantly increased in the peripheral blood serum of obese mice induced by an HFD compared to the ND group (Fig. 2T).

Moreover, to correlate the obesity with monocytes, we fed the normal mice with the ND or HFD for 8 weeks. During this fed period, we collected blood samples every two weeks from HFD-fed mice or ND-fed mice. Then the blood samples were subjected to a CBC test by automatic blood analyzer. Our results showed that the proportion of peripheral monocytes in the HFD-fed mice was significantly time-dependently increased during the HFD-fed period, while there was no significant change for ND-fed mice during the ND-fed (chow-fed) period (Fig. 2U and Supplementary Fig. 2L). These results rule out the possibility that repeated blood samples itself might be responsible for stimulating increase in blood monocytes. Mechanistically, we determined the expression of purine-rich box 1 (PU.1), the myeloid lineage-specific transcription factor and the major regulator of monocytic differentiation [16, 17]. Consequently, we found that mRNA level of PU.1 in HSCs from the HFD group was significantly higher than that in the ND group (Fig. 2V). These findings indicate that HFD-induced obesity may promote the development of monocyte in vivo, which correlates with peripheral dyslipidemia, especially increased NEFA.

### Adipocyte OGT contributes to the monocyte increase in HFD-induced obesity

**Fig. 3:**
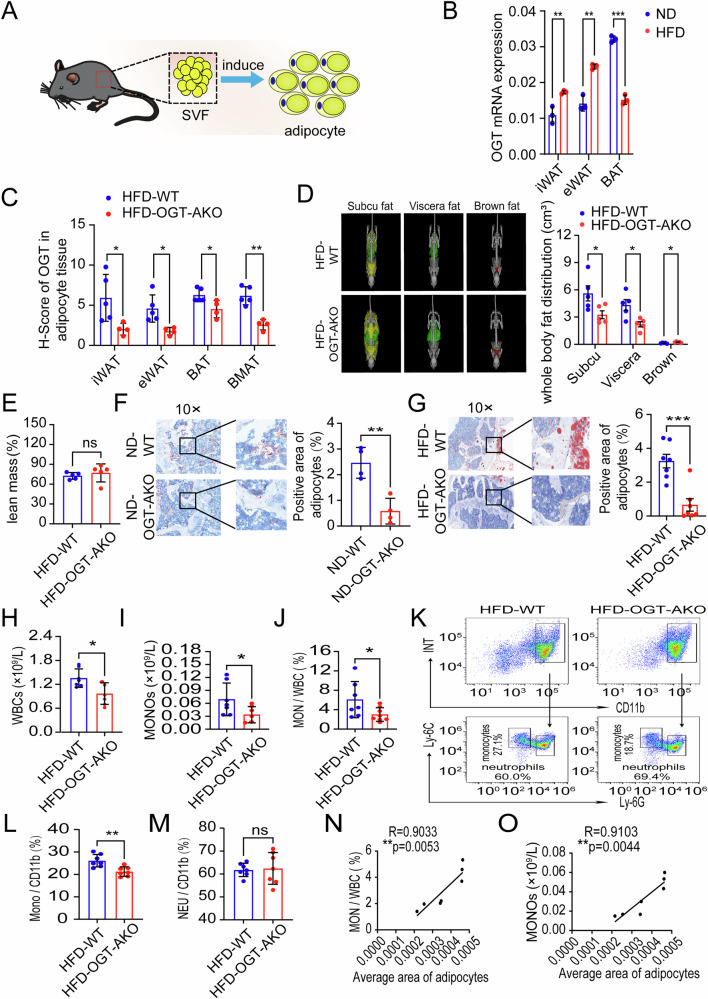
Adipocyte OGT contributes to the monocyte increase in HFD-induced obesity. **A** Schematic diagram of mature adipocytes induced from SVF cells. **B** The mRNA level of OGT in different part of body in adipocytes in ND and HFD groups. **C** The H-Score of OGT expression in iWAT, eWAT, BAT and bone marrow adipose tissue (BMAT) in HFD-WT (*n* = 5) and HFD-OGT-AKO (*n* = 4) groups. **D** Micro CT images of adipose tissue from HFD-WT (*n* = 5) and HFD-OGT-AKO (*n* = 5) group. And the statistical histograms were shown on the right. Yellow represents subcutaneous fat, green represents visceral fat, and red represents brown fat. **E** Lean mass of HFD-WT (*n* = 5) and HFD-OGT-AKO (*n* = 5) mice. **F** ORO staining and quantification of BMAds from ND-WT (*n* = 4) and ND-OGT-AKO (*n* = 4) groups. And the statistical histograms were shown on the right. **G** ORO staining and quantification of BMAds from HFD-WT (*n* = 7) and HFD-OGT-AKO (*n* = 7) groups. And the statistical histograms were shown on the right. **H**–**J** Statistics of absolute value of WBC, MONO and MONO/WBC proportion in PB from HFD-WT (*n* = 7) and HFD-OGT-AKO (*n* = 7) groups. **K**–**M** The MONO and NEU proportion analysis of BM in HFD-WT (*n* = 7) and HFD-OGT-AKO (*n* = 7) groups was shown. And the statistical histograms were shown on the right. **N** Correlation analysis of proportions of MONO/WBC with average area of adipocytes. **O** Correlation analysis of absolute values of MONO with average area of adipocytes. Data were presented as mean ± s.e.m. *p < 0.05, **p < 0.01, ***p < 0.001, ****p < 0.0001, ns no significance, SVF stromal vascular fraction cells.

Given the established link between obesity and elevated monocyte levels, it becomes crucial to explore the role of adipocytes in this process. As OGT has been reported to regulate intracellular lipid metabolism in adipocytes [11, 18], and abnormal OGT signaling can lead to myeloid cell abnormalities [19], we speculate that OGT might mediate the effects of adipocytes on monocytes development. Therefore, we extracted adipose tissue, isolated and induced stromal vascular fraction cells into mature adipocytes (Fig. 3A). By using RT-qPCR and western blot assays, we found that the mRNA and protein levels of OGT in white adipose tissues (iWAT and eWAT) were significantly higher in the HFD group compared to chow-fed (normal diet, ND) group, whereas they were reduced in BAT (Fig. 3B and Supplementary Fig. 3A). These findings indicate that adipocyte OGT expression is significantly increased in white adipocytes of obese mice fed with HFD.

To further explore the role of adipocyte-expressed OGT in obesity, we applied adipocyte-specific OGT knockout (OGT-AKO) mice along with wild-type (WT) mice. OGT-AKO and WT mice were fed with a HFD for 8 weeks. After sacrificing the mice, adipose tissue was isolated and OGT expression was detected. First, we assessed OGT expression in iWAT, eWAT, BAT and bone marrow adipocyte tissue (BMAT) through immunohistochemical staining complemented with HE staining, and the results showed that OGT expression in the HFD-OGT-AKO group was markedly reduced compared to that in the HFD-WT group (Fig. 3C and Supplementary Fig. 3B). Then, the western blot result showed that no OGT was expressed in iWAT, eWAT, and BAT from HFD-OGT-AKO mice compared with normal OGT expression in HFD-WT mice (Supplementary Fig. 3C), confirming the specific knockout of OGT in adipocytes. Next, we investigated the effect of adipocyte OGT knockout on the obesity, and the results showed that the percentage of eWAT weight to body weight in HFD-OGT-AKO mice was significantly lower than that in HFD-WT mice (Supplementary Fig. 3D). Moreover, as for the alteration of adiposity in ND-OGT-AKO mice, we found that the percentage to body weight of eWAT in ND-OGT-AKO mice was significantly reduced compared to that in ND-WT control mice, while no significant change was found for BAT or iWAT (Supplementary Fig. 3E). Additionally, we also found that the weight of HFD-OGT-AKO mice was significantly lower than that of HFD-WT group (Supplementary Fig. 3F). Moreover, we performed a whole-body scan using the microCT Quantum GX2 and observed that the subcutaneous and visceral white adipose tissue volumes in HFD-OGT-AKO mice were significantly reduced compared to those in HFD-WT mice (Fig. 3D). No significant difference of lean mass was found between HFD-WT or HFD-OGT-AKO mice (Fig. 3E). Then, we isolated leg bone of mice and stained adipocytes by using hematoxylin and eosin (H&E) and Oil Red O stains. We observed that bone marrow adipose tissue was reduced in the ND-OGT-AKO group compared to the ND-WT group (Fig. 3F). However, HFD induced a more dramatic decrease in bone marrow adipose tissue in HFD-OGT AKO mice, compared with HFD-WT controls. (Fig. 3G and Supplementary Fig. 3G). All these findings suggest that adipocyte OGT contributes to HFD-induced obesity.

To clarify whether adipocyte OGT was associated with hematopoietic abnormalities in obesity, we first conducted a peripheral CBC analysis, and found that WBC count was significantly reduced in the HFD-OGT-AKO group compared to the HFD-WT group, with no statistical change observed in RBC or PLT (Fig. 3H and Supplementary Fig. 3H, I). In the analysis of WBC subsets, the absolute value or proportion of monocytes significantly decreased, while other cell types showed no significant change (Fig. 3I, J and Supplementary Fig. 3J–M). Compared to the ND-WT control group, no significant difference was observed in the monocyte ratio in the peripheral blood of ND-OGT AKO mice (Supplementary Fig. 3N). Moreover, we determined the expression levels of OGT in monocytes in HFD or ND group mice by flow cytometry. Our results showed that there was no significant difference between ND-OGT-AKO and ND-WT mice (Supplementary Fig. 3O). Additionally, no notable variation in OGT expression in monocytes was found between HFD-OGT-AKO and HFD-WT mice (Supplementary Fig. 3P). These findings suggest adipocyte OGT is associated with the increased peripheral monocytes. We further analyzed the proportions of monocytes or neutrophils in the murine BM from the HFD-WT and HFD-OGT-AKO groups, and found that the proportion of monocytes was significantly reduced, whereas that of neutrophils showed no apparent change (Fig. 3K–M). Furthermore, we correlated the adipocytes and monocytes in the bone marrow. Importantly, after quantifying bone marrow adipose tissue and correlating it to monocyte, we found that the adipose tissue was positively correlated to the absolute value or proportion of monocytes in HFD-OGT-AKO group (Fig. 3N, O). Together, these results indicate that adipocyte OGT contributes to the increased monocytes in HFD-induced obesity.

### Adipocyte OGT facilitates differentiation of hematopoietic stem cells into mature monocytes

**Fig. 4:**
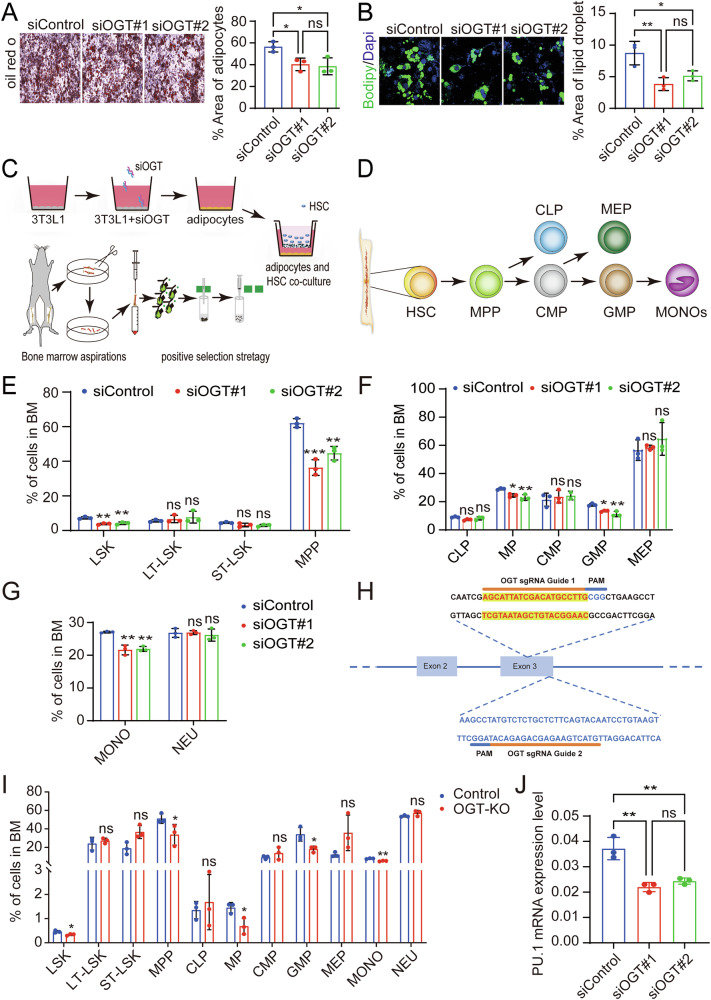
Adipocyte OGT facilitates differentiation of hematopoietic stem cells into mature monocytes. **A** ORO staining and quantification of BMAds from siControl and siOGT group. And the statistical histograms were shown on the right. Data reported as from *n*  = 3 independent biological replicates. **B** BODIPY 493/503 staining and quantification of BMAds from siControl and siOGT group. And the statistical histograms were shown on the right. Data reported as from *n*  = 3 independent biological replicates. **C** Schematic drawing adipocytes and HSCs were co-cultured in transwell. **D** Schematic drawing hematopoietic stem cell differentiation. **E** The statistical histogram of LSKs, LT-LSKs, ST-LSKs, and MPPs proportion of BM in mice. Data reported as from *n*  = 3 independent biological replicates. **F** The statistical histogram of MP, GMP, MEP, CLP and CMP proportion of BM in mice. Data reported as from *n*  = 3 independent biological replicates. **G** The statistical histogram of MONO and NEU proportion of BM in mice. Data reported as from *n*  = 3 independent biological replicates. **H** Schematic of the sgRNAs targeting OGT in 3T3L-1 cells. The sgRNAs were designed for KO targeting the third exon of OGT gene. **I** The statistical histogram of hematopoietic stem cells and their subgroups of cells of BM in mice. Data reported as from *n*  = 3 independent biological replicates. **J** The mRNA levels of PU.1 of LSKs in siControl groups and siOGT groups. Data reported as from *n*  = 3 independent biological replicates. Data were presented as mean ± s.e.m. *p < 0.05, **p < 0.01, ***p < 0.001, ****p < 0.0001, ns no significance.

To elucidate the regulatory role of adipocyte OGT in monocyte development, we initiated experiments by silencing OGT using siRNA in 3T3-L1 pre-adipocytes, which were then induced to differentiate into mature adipocytes. The results indicated that mRNA and protein levels of OGT were reduced in mature adipocytes (Supplementary Fig. 4A, B). Following Oil Red O staining, the adipose in OGT- downregulated cells (siOGT) was significantly reduced compared to the control (siControl) (Fig. 4A). Lipid droplet accumulation and content were assessed by using Bodipy staining, and the results showed that the area of lipid droplets in siOGT cells was also significantly reduced (Fig. 4B). These findings support that knocking down OGT expression in the 3T3-L1 cells reduces the production of mature adipocytes.

Next, to explore the role of adipocyte OGT in HSC differentiation and monocyte development, Lin (−) Sca1(+) c-Kit (+) (LSK) cells comprising multipotent HSCs were isolated from murine bone marrow and co-cultured with OGT-downregulated adipocytes by using a transwell-based co-culture system (Fig. 4C). Monocyte development consists of several myeloid stages initiated by multipotent, self-renewing HSCs in the bone marrow (Fig. 4D). After 24 h of co-culture, the flow cytometry results showed that the proportions of LSKs and multipotent progenitors (MPPs) were significantly reduced while no significant change in long term (LT) LSKs and short term (ST) LSKs when being co-cultured with siOGT adipocytes compared those with siControl adipocytes (Fig. 4E and Supplementary Fig. 4C). Additionally, compared to the siControl group, the proportion of myeloid progenitor cells (MPs) or granulocyte-monocyte progenitors (GMPs) in siOGT decreased significantly, while there was no statistical change in other progenitor cell pools, including common lymphoid progenitors (CLPs), common myeloid progenitors (CMPs), and megakaryocyte-erythrocyte progenitors (MEPs) (Fig. 4F and Supplementary Fig. 4D). Furthermore, the proportion of monocytes was apparently decreased, whereas neutrophils showed no significant change (Fig. 4G and Supplementary Fig. 4E). To verify these results, we utilized CRISPR-Cas9 technology to knockout the OGT gene in 3T3-L1 cells and further investigated its effect on the differentiation of HSCs into monocytes (Fig. 4H). Based on the results of knockout efficiency detected by using western blot method (Supplementary Fig. 4F, G), we selected sgOGT#2-transfected monoclonal 3T3L1 cells, namely OGT-KO-3T3-L1, for the following experiments. The selected monoclonal OGT-KO-3T3-L1 cells were amplified and induced into adipocytes (named OGT-KO-adipocytes), which were then co-cultured with HSCs. Then, flow cytometry was performed to determine the differentiation of HSCs. The results showed that the percentage of LSK, MPP, GMP, or monocyte population was significantly reduced when co-cultured with OGT-KO-adipocytes, which corroborates our previous results (Fig. 4I). These results suggest that adipocyte OGT promotes HSCs differentiated into monocytes in vivo.

Furthermore, we applied colony-forming units (CFUs) assay to determine the in vitro effect of adipocyte OGT on HSCs differentiation. The results showed that, compared to the siControl, the number of the CFU- granulocyte/monocyte (CFU-GM) or the CFU- monocyte (CFU-M) in siOGT was significantly reduced, indicating that these progenitors are reduced in both number and function (Supplementary Fig. 4H). Furthermore, the mRNA level of PU.1 in LSKs was significantly decreased in siOGT group (Fig. 4J). Altogether, these data suggest that adipocyte OGT promotes monocyte development at an early stage of HSC differentiation.

### Regulatory role of NEFA in adipocyte OGT-mediated monocyte increase in obesity

**Fig. 5:**
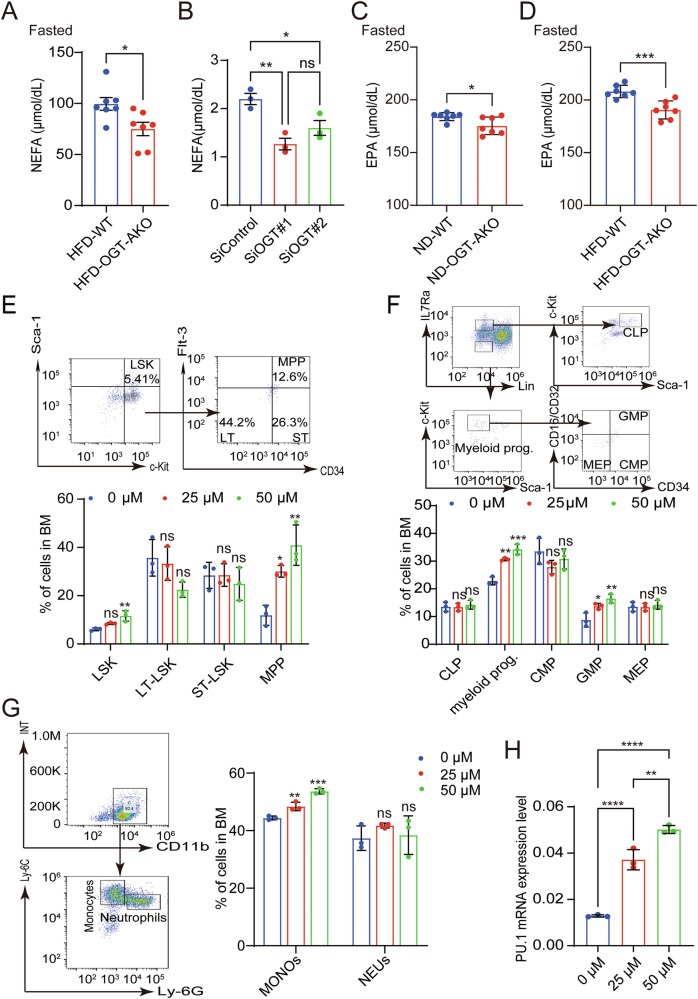
Regulatory role of NEFA in adipocyte OGT-mediated monocyte increase in obesity. **A** The serum level of NEFA in HFD-WT (*n* = 7) and HFD-OGT-AKO (*n* = 7) groups in 12 h fasted state. **B** The serum level of NEFA in siControl and siOGT groups. Data reported as from *n*  = 3 independent biological replicates. **C** The serum level of EPA in ND-WT (*n* = 7) and ND-OGT-AKO (*n* = 7) groups in 12 h fasted state. **D** The serum level of EPA in HFD-WT (*n* = 7) and HFD-OGT-AKO (*n* = 7) groups in 12 h fasted state. **E** The LSKs, LT-LSKs, ST-LSKs, and MPPs proportion analysis of BM in mice after being treated with 0, 25, 50 μmol/L EPA for 24 h was shown. And the statistical histogram was showed below. Data reported as from *n*  = 3 independent biological replicates. **F** The Myeloid progenitor cells, GMP, MEP, CLP and CMP proportion analysis of BM in mice was shown. And the statistical histograms were shown on the right. Data reported as from *n*  = 3 independent biological replicates. **G** The MONO and NEU proportion analysis of BM in mice was shown. And the statistical histograms were shown on the right. Data reported as from *n*  = 3 independent biological replicates. **H** The mRNA levels of PU.1 of HSCs in mice after being treated with 0, 25, 50 μmol/L EPA for 24 h was shown. Data reported as from *n*  = 3 independent biological replicates. Data were presented as mean ± s.e.m.*p  < 0.05, **p < 0.01, ***p < 0.001, ****p < 0.0001, ns no significance.

As shown above that increased NEFA was positively correlated with increased monocytes in obesity and adipocyte OGT promotes monocytes development, we wondered whether NEFA could mediate the regulated effect of adipocyte OGT on monocyte. We first determined the level of peripheral NEFA and found that serum NEFA level was significantly lower in the HFD-OGT-AKO group while no statistical change was found for other indices compared with HFD-WT group (Fig. 5A and Supplementary Fig. 5A–E). In vitro, we further collected cell supernatant to measure NEFA level and found that NEFA level in the siOGT group was lower than that in the siControl group (Fig. 5B). Therefore, these findings suggest that NEFA may be involved in the regulated effects of adipocyte OGT on monocytes development.

Eicosapentaenoic acid (EPA), a principal component of NEFA, has been shown to increase the monocyte IL-10 expression and have an effect on blood mononuclear cells [20]. We first measured the EPA level in human peripheral blood, and found no significant difference between the underweight group, normal weight group and overweight group (Supplementary Fig. 5F; Table 2). We further determined the levels of EPA in ND and HFD mice. Our results showed that the peripheral blood level of EPA had increased trend in HFD group, but did not reach statistical difference (Supplementary Fig. 5G). We also measured EPA levels in WT and OGT-AKO mice under HFD or ND feeding condition. The results revealed that the EPA level was significantly reduced in OGT-AKO mice compared to WT mice under ND condition, and decreased much more significantly under HFD condition (Fig. 5C, D). These findings suggest that OGT-AKO mice exhibit impaired EPA metabolism, which might be exacerbated under high-fat diet condition.

**Table 2:** Clinical characteristics and metabolic markers of subjects.

Clinical indication	underweight people (*n* = 57)	Normal range (*n* = 883)	overweight people (*n* = 731)	*p* value
Age (year)	21–67	19–93	20–88	<0.0001
Gender (Male/Female)	12/45	335/548	544/187	<0.0001
BMI	17.62 ± 0.73	22.29 ± 1.73	27.92 ± 2.56	<0.0001
WBC (*10^9^/L)	5.59 ± 1.39	5.93 ± 1.43	6.53 ± 1.54	<0.0001
RBC (*10^12^/L)	4.58 ± 0.38	4.68 ± 0.46	5.01 ± 0.43	<0.0001
HGB (g/L)	135.02 ± 16.29	138.88 ± 17.18	150.32 ± 14.58	<0.0001
PLT (*10^9^/L)	263.82 ± 55.52	263.59 ± 60.56	263.38 ± 58.79	0.9967
NEU (*10^9^/L)	3.25 ± 1.10	3.47 ± 1.11	3.79 ± 1.13	<0.0001
LYM (*10^9^/L)	1.91 ± 0.53	1.97 ± 0.53	2.17 ± 0.64	<0.0001
MONO (*10^9^/L)	0.30 ± 0.09	0.33 ± 0.11	0.38 ± 0.11	<0.0001
EOS (*10^9^/L)	0.10 ± 0.07	0.13 ± 0.12	0.16 ± 0.15	<0.0001
BAS (*10^9^/L)	0.03 ± 0.01	0.03 ± 0.02	0.03 ± 0.02	0.0002
NEU%	57.32 ± 8.18	57.89 ± 7.93	57.61 ± 7.54	0.7082
LYM%	34.82 ± 8.03	33.73 ± 7.37	33.59 ± 7.18	0.4779
MON%	5.53 ± 1.28	5.65 ± 1.45	5.87 ± 1.42	0.0018
EOS%	1.84 ± 1.17	2.21 ± 1.90	2.42 ± 1.99	0.0103
BAS%	0.50 ± 0.23	0.51 ± 0.28	0.51 ± 0.28	0.9242
TC (mmol/L)	4.46 ± 0.84	4.74 ± 0.91	4.95 ± 0.90	<0.0001
TG (mmol/L)	0.91 ± 0.56	1.22 ± 1.08	1.87 ± 1.60	<0.0001
HDL (mmol/L)	1.64 ± 0.33	1.41 ± 0.32	1.22 ± 0.25	<0.0001
LDL (mmol/L)	2.23 ± 0.66	2.71 ± 0.71	2.97 ± 0.72	<0.0001
FBG (mmol/L)	4.49 ± 0.46	4.77 ± 1.0	5.13 ± 1.34	<0.0001
NEFA (μmol/dL)	32.8 ± 8.58 (*n* = 10)	40.8 ± 8.04 (*n* = 55)	47.4 ± 7.86 (*n* = 36)	<0.0001
EPA (pg/mL)	340.74 ± 200.51 (*n* = 10)	363.95 ± 458.63 (*n* = 55)	386.91 ± 309.30 (*n* = 36)	0.1761

*BMI* body mass index, *WBC* white blood cell, *RBC* red blood cell, *HGB* Hemoglobin, *PLT* platelet, *NEU* Neutrophil, *LYM* lymphocyte, *MONO* monocytes, *EOS* eosinophil, *BAS* basophils, *TC* total cholesterol, *TG* triacylglycerol, *HDL* high-density lipoprotein cholesterol, *LDL* low-density lipoprotein cholesterol, *GLU* glucose, *NEFA* nonesterified fatty acids, *EPA* eicosapentaenoic acid.

Next, we applied EPA to the culture system of murine LSKs, and determined the hematopoietic lineage differentiation by FACS. Our results showed that EPA treatment significantly increased the proportions of LSKs and the myeloid-associated stem and progenitor cell pools, including MPPs, myeloid progenitor cells, and GMPs, except for MEPs, CLPs, and CMPs (Fig. 5E, F). Furthermore, we determined the bone marrow terminal differentiation cells, and found that EPA significantly increased the proportion of monocytes at a concentration-dependent manner while neutrophils remained unchanged (Fig. 5G). Additionally, the mRNA expression levels of PU.1 in HSCs were also restored by addition of EPA (Fig. 5H). Therefore, our data indicated that in obesity, increased adipose-derived NEFA’s promote HSC and monocyte development.

### Adipocyte OGT modulates monocyte development via NEFA-CD36/FABP4 signaling pathway

**Fig. 6:**
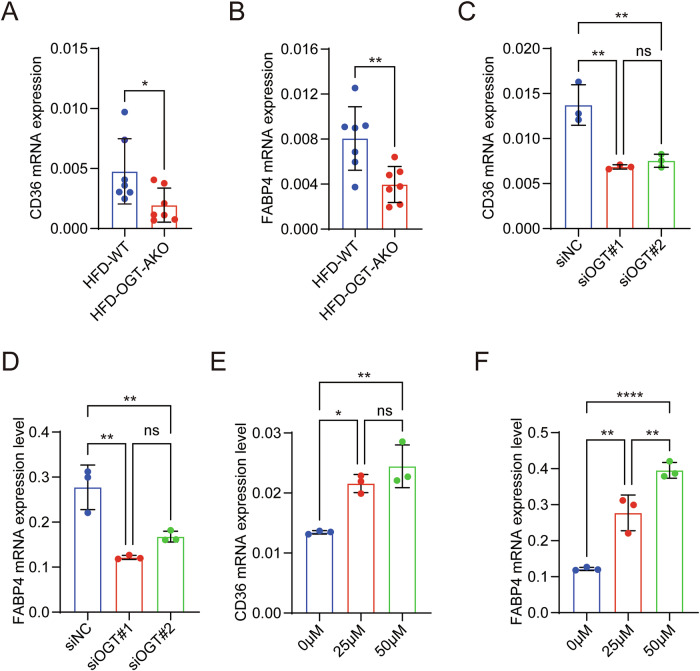
Adipocyte OGT modulates monocyte development via NEFA-CD36/FABP4 signaling pathway. **A**, **B** The mRNA levels of CD36 and FABP4 of HSCs in HFD-WT (*n* = 7) and HFD-OGT-AKO (*n* = 7) groups. **C**, **D** The mRNA levels of CD36 and FABP4 of HSCs in siControl groups and siOGT groups. Data reported as from *n*  = 3 independent biological replicates. **E**, **F** The mRNA levels of CD36 and FABP4 of HSCs in mice after being treated with 0, 25, 50 μmol/L EPA for 24 h was shown. Data reported as from *n*  = 3 independent biological replicates. Data were presented as mean ± s.e.m. *p  < 0.05, **p < 0.01, ***p < 0.001, ****p < 0.0001, ns no significance.

Adipocyte OGT plays a pivotal role in regulating monocyte development through modulation of NEFA levels and their intracellular pathways. Cluster of differentiation 36 (CD36), a receptor known to facilitate the uptake of long-chain fatty acids into HSCs, is crucial for their internalization and utilization, directly impacting cellular energy and signaling pathways [21]. And fatty acid binding proteins (FABPs) are also reported be crucial for intracellular fatty acid transport [22, 23]. To explore the mechanism of NEFA in adipocyte OGT-induced monocyte development, we then determined the mRNA expression levels of CD36 and FABP4 in HSCs. Real-time qPCR analysis revealed that OGT deficiency in adipocytes led to significantly decreased mRNA level of CD36 or FABP4 in HSCs in the HFD-OGT-AKO mice compared to that in the HFD-WT mice (Fig. 6A, B). Remarkably decreased mRNA expression levels of CD36 and FABP4 were also observed in co-cultured HSCs in the siOGT group compared to those in the siControl group (Fig. 6C, D). In a NEFA rescue experiment, we observed that the mRNA expression levels of CD36 and FABP4 in HSCs were gradually increased at a concentration-dependent manner in the co-culture system (Fig. 6E, F). Therefore, adipocyte OGT regulates serum NEFA levels, which in turn stimulates the upregulation of CD36 and FABP4 mRNA expression, facilitating their uptake in HSCs and ultimately leading to an increased formation of monocytes.

## Discussion

Obesity has become a significant health concern due to its strong association with various diseases, including cardiovascular disease, type 2 diabetes, hyperlipidemia, hypertension, and malignancy [24]. The development of hematopoietic cells occurs in a gradual and hierarchical manner. There are long-term and short-term forms of HSCs, each differing in their ability to self-renew and differentiate. Long-term HSCs (LT-HSCs), located in the early stages of differentiation, are utilized to meet the enormous daily hematopoietic demand while maintaining the lifetime activity of the hematopoietic niche [25]. LT-HSCs are activated solely by extracellular signals, and subsequently produce cells with strong proliferative capacity but short life spans, including short-term HSCs (ST-HSCs) and MPPs. ST-HSCs have limited self-renewal ability, and differentiate into MPPs. MPPs have no self-renewal capabilities, but retain the potential to differentiate into CMPs and CLPs. Both CMPs and CLPs belong to early hematopoietic progenitor cells, which still have strong proliferation and multipotent differentiation ability. CMPs are further differentiated into MEPs and GMPs. MEPs are hematopoietic progenitor cells of platelets, which eventually differentiate into platelets. Erythroid progenitors are erythroid progenitors in the BM that eventually differentiate into red blood cells. GMPs eventually differentiate into eosinophils, neutrophils, basophils, and monocytes. CLPs are differentiated to committed progenitor T cells and progenitor B cells that ultimately become mature T cells and mature B cells [26, 27]. HSCs produce all mature hematopoietic cells, maintaining their function and quantity to preserve the homeostasis of the hematopoietic system [4].

Currently, the role of obesity in HSC differentiation remains controversial. Studies have confirmed that an increase in adipocytes in the thoracic region and a reduction in HSC and ST-HSC production in adipocyte-rich murine bone marrow [5]. Research verifies that, irreversible destruction of HSC hematopoietic function by mouse BMAds, mediated by disruptions in the lipid raft/TGF-β signaling pathway, even after bone marrow transplantation [28]. Other studies argue against this view. Following whole-body irradiation, the bone marrow microenvironment, including endothelial and mesenchymal stem cells, is temporarily disrupted. In response, MSCs rapidly differentiate into adipocytes, which temporarily substitute for the HSC niche and maintain basic hematopoietic functions by secreting stem cell factor [29]. Thus, studying how obesity influences hematopoietic cell formation and its underlying molecular mechanisms provides crucial theoretical insights for diagnosing and treating hematological diseases. Previous studies have demonstrated that HBP-OGT signaling regulates leptin expression in adipose tissue in response to nutrient availability [30, 31]. Herein, we found that BMI independently associates the development of hematopoietic cells, particularly monocytes, as evidenced by analyzing large-scale clinical data. Using obese mouse models and adipocyte-specific OGT gene knockout mice fed with HFD, we demonstrated that obesity leads to changes in serum lipid profiles and promotes monocyte development via the up-regulation of OGT gene expression in adipocytes.

Lipids play a crucial role in regulating cellular homeostasis, encompassing cellular signaling, transport, immunity, maintenance of cell structure, and metabolism [32–34]. Bone marrow adipocytes can decrease bone mass by inhibiting osteoblast differentiation, subsequently altering hematopoietic function [35–37]; however, the specific molecular mechanisms remain poorly understood. To explore the molecular mechanisms by which adipocytes regulate monocyte formation, we utilized the HFD-OGT-AKO mouse model and observed a significant reduction in white adipocytes. Human adipose tissue has a remarkable capacity for lipid storage. When subjected to lipolysis, it releases lipid mediators that can lead to insulin resistance and the accumulation of fatty acids in organs such as the heart, pancreas, liver, blood vessels, and kidneys [38]. Disruptions in adipose tissue lipid metabolism can elevate plasma NEFA concentration, leading to ectopic fat deposition [39]. Dyslipidemia results in adipose tissue dysfunction, increased serum NEFA level, and imbalance between pro-inflammatory and anti-inflammatory adipokines [40]. In this study, knockout of the adipocyte OGT significantly reduced serum NEFA level in HFD mice. EPA is one of the important components of NEFA. When EPA exists in free form, it belongs to one of the free fatty acids and is mainly used in clinical treatment of dyslipidemia. It has been previously reported that increased intake of EPA affects monocyte function[20, 41]. Our results revealed that the EPA level in OGT-AKO mice was significantly reduced compared to the WT group under ND and HFD conditions, and suggested that OGT-AKO mice exhibit impaired EPA metabolism. HSCs and hematopoietic progenitors rely on fatty acid oxidation as an energy source for self-renewal and differentiation [42]. Cell culture and co-culture experiments of adipocytes with HSCs showed that downregulating OGT expression in adipocytes decreased NEFA content in the co-culture supernatant and reduced the proportions of LSKs, MPPs, myeloid progenitors, GMPs, and monocytes. Thus, adipocytes regulate the differentiation of HSCs into monocytes via the OGT-NEFA pathway both in vivo and in vitro.

**Fig. 7:**
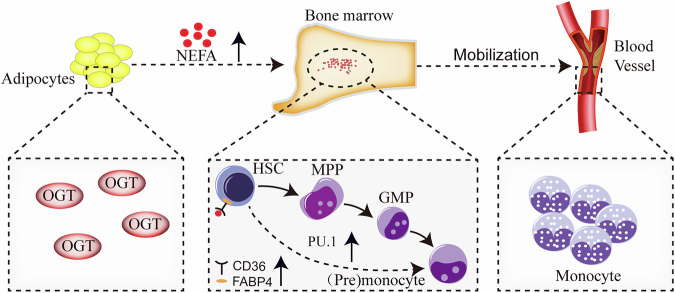
Schematic diagram illustrating the molecular mechanisms of the increased formation of monocytes in obesity.

Obesity results from excessive adipocyte accumulation, which is a fundamental cause of various diseases [43]. Obesity is linked to poorer prognosis in various cancers, including hematologic malignancies [44]. Numerous studies have demonstrated a strong correlation between myeloid leukemia and obesity in both children and adults, indicating increased risk and worse survival outcomes for obese and overweight individuals [45, 46]. However, the relationship between obesity and the growth and survival of leukemia pre-stem and progenitor cells is unclear. Here, we elucidate the molecular mechanisms by which adipocyte OGT promotes the differentiation of HSCs into monocytes in obesity, regulating monocyte formation through the OGT-NEFA-CD36/FABP4 pathway (Fig. 7). Studies have shown that leukemia stem cells (LSCs) interact with their microenvironment, leading to the lipolysis of bone marrow adipocytes, which fuel the LSCs through fatty acid oxidation. LSCs that express the fatty acid transporter CD36 exhibit elevated levels of fatty acid oxidation, conferring a survival advantage to these cells [47]. Previous studies by our team have shown that high expression of OGT is positively associated with poor prognosis in acute myeloid leukemia (AML) [48]. AML is a malignant tumor originating from myeloid stem cells or progenitors. Acute monocytic leukemia, the AML-M5 subtype, is characterized by a significantly increased proportion of naive, primitive monocytes in the bone marrow, impairing normal hematopoietic function [49]. Obesity increases mortality in patients with various cancer subtypes, including leukemia [50]. There is a significant survival difference between lean and obese patients, closely linked to the direct effects of the adipose-enriched microenvironment on cancer cells, although the molecular mechanisms are seldomly reported [50]. As the development of OGT inhibitors is progressing rapidly [51], systematically inhibiting OGT presents a promising strategy to combat obesity-induced hematologic malignancies.

## Materials and methods

### Characteristics of the subjects

A total of 1671 adult subjects medical check-ups were included in this study from February 2021 to August 2022 at Qilu Hospital of Shandong University with a median age of 41 years (19–93 years). Obesity is defined by the World Health Organization (WHO) and by Asian-specific cutoffs for body BMI. The BMI threshold for underweight is less than 18.5 kg/m². According to both Asian and WHO classifications, the BMI cutoff for overweight is 25–29.9 kg/m² and obesity is 30 kg/m² or greater [52]. The classification of overweight/obesity by BMI followed the WHO (2000) classification strictly [53]. Based on their BMI, participants were divided into an underweight group (12 males and 45 females), a normal range group (335 males and 548 females), and an overweight (544 males and 187 females). The basic characteristics of all participants, including age, gender, peripheral blood counts, and serum biochemicals, are presented in Table 2. The study was approved by the Medical Ethical Committee of Qilu Hospital of Shandong University.

### Murine model and treatment

C57BL/6 mice (male, 6–8 weeks old) were purchased from Beijing Vital River Laboratory Animal Technology Company. Mice were fed either a normal diet or a 60% high-fat diet (Research Diets, D12492) for 8 weeks. The standard obesity model utilized in this study involves mice with a body weight exceeding 40 grams. The sample size for each group comprised no fewer than four mice. Animal protocols were approved by the Animal Ethics Committee of Qilu Hospital of Shandong University. Animal experiments were conducted with random grouping to control potential bias; therefore, blinding was not applied. The study was approved by the Medical Ethical Committee of Qilu Hospital of Shandong University.

Ogt-floxed mice (harboring the OGT conditional allele) and adipoq-CreER mice (harboring the adiponectin-CreER) on a C57BL/6 background were obtained from College of Life Sciences of Shandong Normal University and cultured in SPF condition. To induce Cre recombinase activity, mice were injected intraperitoneally (i.p.) with 100 μL tamoxifen (Sigma-Aldrich, 20 mg/ml in vegetable oil) daily for 5 days. Adipose-specific OGT knockout (AKO) mice were generated by crossing Ogt-floxed mice with adipoq-CreER mice. Segments of 3–5 mm from mouse tails or 1–2 mouse toes were clipped and placed in 1.5 mL Eppendorf tubes. Subsequently, DNA was extracted from the mouse tissue, and the genotype was identified. Primers for scrambled OGT-F (5′-CAT CTC TCC AGC CCC ACA AAC TG-3′), OGT-R (5′-GAC GAA GCA GGA GGG GAG AGC AC-3′), Lox P1 (5′-ATT TGC CTG CAT TAC CGG TCG-3′), Lox P2 (5′-CAG CAT TGC TGT CAC TTG GTC-3′), Cre1 (5′-CAA ATG TTG CTT GTC TGG TG-3′), and Cre2 (5′-GTC AGT CGA GTG CAC AGT TT-3′) were synthesized by BioSune Company in Shanghai, China.

### Cell culture

The 3T3-L1 cell line was purchased from Zhongqiao Xinzhou Biotechnology Co., LTD (shanghai, China). The cells have been identified by short tandem repeat (STR) and tested for mycoplasma contamination. The 3T3-L1 cell line was cultured in Dulbecco’s Modified Eagle Medium (DMEM) (Gibco, USA) containing 10% newborn bovine serum (NBS) and 1% penicillin/streptomycin. 3T3-L1 cells were seeded in a culture dish until cell density reached 100%. The medium was replaced once, and differentiation into mature adipocytes was induced after an additional 2 days of growth. Cell medium I was added to induce differentiation, and the cells were cultured for another 2 days. Cell medium I consists of complete medium (DMEM with 10% Gibco fetal bovine serum (FBS)), 0.5 mM IBMX, 1 μM dexamethasone, and 10 μg/mL insulin. Cell medium II was added to further induce differentiation and the cells were cultured for an additional 2 days. Cell medium II consists of complete medium (DMEM with 10% Gibco FBS) and 10 μg/mL insulin. The cell culture medium was changed every 2 days. After approximately 10–12 days, a large number of fat droplets are visible under an ordinary light microscope.

Stromal vascular fraction was isolated from white fat collected from the groin or epididymis of mice. The tissue was diced into 2 mm pieces and placed in a digestive buffer containing sterile phosphate buffered saline (PBS) supplemented with 10 mg/mL collagenase D (Roche, 11088882001), 2.4 mg/mL dispase II (Roche, 04942078001), and 1 mM CaCl_2_, then incubated at 37 °C while shaking at 140 rpm for 40 min. Periodically, tissues were briefly vortexed and returned to continue digestion. Post-digestion, tissues were filtered through a 100 μm sterile filter, washed with 10 mL of cell medium to inactivate the collagenase, and centrifuged at 4 °C, 500 × *g* for 10 min. The supernatant was discarded, and the pellet was resuspended in 10 mL of medium, vigorously pipetted up and down at least 10 times to achieve a single-cell suspension. This suspension was then filtered through a 70 μm membrane, centrifuged under the same conditions, and the cells were resuspended in medium, pipetted again, and plated on collagen-coated cell culture plates. After 24 h of culture, cells were washed with PBS and vigorously shaken to remove debris. Once cell density reached 100% confluence, differentiation was induced using a medium supplemented with 0.5 mM IBMX, 1 μM dexamethasone, 850 nM insulin, and 1 μM rosiglitazone. After 48 h, the medium was switched to contain only 850 nM insulin and 1 μM rosiglitazone. Rosiglitazone was removed on day 4, but insulin treatment continued. The insulin-only medium was refreshed on day 6. By day 7, the differentiation of cells was confirmed successful.

A transwell chamber, matching the pore plate used for the cultured mature adipocytes, was selected. Cells sorted by magnetic beads were then transferred to a transwell chamber with a pore size of 0.4 μm. The transwell chamber was positioned in a well of the culture plate to co-culture with mature adipocytes.

### siRNA transfection experiment

In a cell culture plate with a cell density of 30–50%, cells were treated with RNAiMAX (Invitrogen, 13778030) and siRNA (Biosune, shanghai) in serum-free medium for 4–6 h. After this incubation, the medium was replaced, and the cells were cultured for an additional 48 h before being used for subsequent experiments.

### Flow cytometry

At the designated time, mice were euthanized, and bone marrow cells were harvested and dissociated into single cells. Co-cultured cells were transferred from the transwell chamber to a flow tube and centrifuged at 1500 rpm for 5 min. The cells were then washed once with an appropriate volume of PBS and centrifuged again at 1500 rpm for 5 min. Each sample was resuspended in PBS, and different flow cytometry antibodies were added sequentially. Cell surface staining was performed using specific markers at 4 °C for 30 min. After staining, the cells were washed with PBS to remove unbound antibodies and centrifuged at 1500 rpm for 5 min, discarding the supernatant afterward. A final volume of 300 µL PBS was added to each cell suspension for immediate analysis by flow cytometry. The voltage was adjusted to maximize the separation between positive and negative cell populations. The expression of OGT in mouse monocytes was detected using an indirect labeling method. Specifically, mononuclear cells were isolated from mice according to the previously described protocol. The cells were subsequently fixed and permeabilized using the FIX & PERM Kit (LinkBio, GAS006). Next, the cells were incubated with a primary OGT antibody at 4 °C for 1 h. Following incubation, the cells were washed once with PBS. A fluorescent secondary antibody conjugated to APC was then added, and the cells were incubated again at 4 °C for 1 h. After another wash with PBS, the cells were resuspended in 300 µL of buffer and analyzed the ratio of CD11b^+^Ly6C^high^ cells by flow cytometry. Detailed information on the flow cytometry antibodies used is listed in Table 3. Cell analysis was conducted using a Gallios flow cytometer (Beckman Coulter, USA), and the data were analyzed using FlowJo software.

**Table 3:** Key antibodies used in flow cytometry.

Antibodies	Channel	Source	Identifier
Anti-mouse lineage cocktail (B220, CD3e, CD11b, Gr1, Ter119)	FITC	Biolegend	133301
Anti-mouse Sca1	PE-Cyanine7	eBioscience	25-5981-82
Anti-mouse C-kit	PE	eBioscience	12-1171-81
Anti-mouse Flt-3	APC	eBioscience	17-1351-82
Anti-mouse CD34	APC-700	eBioscience	56-0341-82
Anti-mouse IL7Ra	APC	Biolegend	135011
Anti-mouse CD16/CD32	PE-Cyanine5.5	eBioscience	45-0161-80
Anti-mouse CD11b	PE-Cyanine7	Biolegend	101216
Anti-mouse Ly-6C	PE	eBioscience	12-5932-82
Anti-mouse Ly-6G	APC	Biolegend	127614

*FITC* Fluorescein Isothiocyanate, *PE* Phycoerythrin, *APC* Allophycocyanin.

### Real-time quantitative PCR

Total RNA was extracted from cells using Trizol (Invitrogen, USA) and subsequently reverse-transcribed into cDNA using the PrimeScript RT Reagent Kit Perfect Real Time (Takara Bio, Japan). Quantitative PCR was performed on the LightCycler 480 II real-time fluorescent quantitative PCR system (Roche, Switzerland). Primer sequences are listed in Table 4. The experiment was repeated three times to ensure reliability.

**Table 4:** Summary of PCR primer sequences.

Primer	Sequences (5’ → 3’)
Mus-Ogt-137F	AAGCTCAGTGATGGCCGATT
Mus-Ogt-137R	GGGCTCAAGGCATAGCAGAA
Mus-PU.1-107F	CAGCAGCTCTATCGCCACAT
Mus-PU.1-107R	ATCCGGGGCATGTAGGAAAC
Mus-Fabp4(422/ap2)-90F	TCACCATCCGGTCAGAGAGTA
Mus-Fabp4(422/ap2)-90R	TCCTGTCGTCTGCGGTGATT
Mus-Cd36-77F	GATCGGAACTGTGGGCTCAT
Mus-Cd36-77R	ACTGGCATGAGAATGCCTCC
Mus-GAPDH F	GTTCCTACCCCCAATGTGTCC
Mus-GAPDH R	TAGCCCAAGATGCCCTTCAGT

*Mus* Mouse.

### Western blot assay

Cells were collected and lysed using a total protein extraction kit (Bestbio, China). Protein concentration was determined using a BCA protein assay kit. Equal amounts of each protein sample were mixed with loading buffer, loaded onto a 10% SDS-PAGE gel, and electrotransferred onto a 0.2 μm PVDF membrane. After blocking at room temperature for 60 min, the PVDF membrane was incubated with OGT antibody (ab96718, Abcam, USA) and GAPDH antibody (AB0038, Abways Technology, China) overnight at 4 °C. Detection was performed using the SageCapture chemiluminescence imaging system (ChampChemi 610 Plus, China) after coupling with secondary antibodies.

### Complete blood count and blood biochemical indexes assay

For complete blood count measurements, blood was collected from the tail vein at specified times and analyzed using the Mindray BC-7500CS automatic blood analyzer. For the determination of blood biochemical indexes including nonesterified fatty acids (NEFA), peripheral blood was collected at the time of mice sacrifice, and the assays were measured using the Roche cobas8000 automatic biochemical analyzer.

### Analysis of bodipy 493/503 staining

Initially, 3T3-L1 cells were treated with siRNA (SiControl and SiOGT) for 48 h. Subsequently, we induced them differentiated into mature adipocytes. Cells were incubated at 37 °C for 30 min with 1 μmol/L BODIPY 493/503 (Shanghai Maokang Biotechnology, China). After being washed twice with PBS, the cells were counterstained with 4’,6-Diamidino-2-phenylindole (DAPI) and mounted on slides for confocal microscopy analysis. Average fluorescence intensity was measured.

### Oil red o staining analysis in adipocytes

Following the standard protocol of the Oil Red O Staining Kit (Bestbio, China), the cell medium was first removed, and the cells were washed twice with PBS. They were then fixed using Oil Red O Fixative A for 25 min. After fixation, the cells were soaked in 60% isopropyl alcohol for 5 min, which was subsequently discarded. A freshly prepared mixture of Oil Red O staining solutions B1 and B2 was then added for a 15 min soak. Following this, the staining solution was discarded, and the cells were washed six times to remove any excess dye. Mayer’s hematoxylin staining solution C was applied to re-stain the nuclei for 2 min. After discarding the hematoxylin stain, the cells underwent another six washes, followed by the addition of Oil Red O buffer for 1 min before discarding. Lastly, distilled water was added to cover the cells, and they were observed under a microscope.

### Immunohistochemistry and hematoxylin-eosin staining

Immunohistochemistry and hematoxylin-eosin staining were performed on the tissue samples (adipocyte tissue and decalcified bone tissue) to accurately assess the expression level of OGT. The slides were deparaffinized, antigen repaired, blocked of endogenous peroxidase and non-specific binding sites. Then, the slides were sealed with 3% bovine serum albumin (BSA) at room temperature for 30 min and then incubated with primary antibody of OGT (ab96718, Abcam, USA) at 4 °C for 50 min. Next, the sections were incubated with secondary antibody (GB23303, Servicebio, China) for 1 h and diaminobenzidine (DAB) for 3 min, followed by hematoxylin staining. Subsequently, the sections were subjected to eosin staining, then dehydrated and sealed in neutral resin. The sections were scanned using 3DHISTECH Pannoramic MIDI II and analyzed using Servicebio Alpathwell v2. In the stained sections, nucleus appears blue, and cytoplasm appears red and OGT protein appears brown.

Histochemistry score (H-score) parameters were utilized to analyze the immunohistochemical results. The H-score is calculated as follows: ∑(pi × i), where pi represents the percentage of cells at each staining intensity level, and i indicates the grade classification of positive cells. Specifically: Negative (no staining): 0 point; Weak positive (light yellow): 1 point; Moderate positive (brown-yellow): 2 points; Strong positive (tan): ≥3 points.

### Colony formation assay

Thaw MethoCult GF M3534 medium at room temperature and vigorously vortex to ensure thorough mixing. Allow the medium to stand until bubbles dissipate. Inoculate each well of a 24-well plate with 2 × 10^4^ co-cultured hematopoietic stem cells. Vortex the centrifuge tube again to ensure even distribution of cells and allow time for any bubbles to dissipate. Using a syringe equipped with a sterile 16-gauge needle, carefully transfer the cell-containing culture medium into the wells of the 24-well plate, ensuring each condition (control and experimental) was replicated in three wells. Place the plates in a cell incubator and allow colonies to form over a period of 14 days, after which colonies can be identified and counted.

### Whole-body quantitative micro-computed tomography (microCT) scan

To assess the accumulation and distribution of adipose tissues including subcutaneous, visceral and brown fat, whole-body micro-computed tomography (microCT Quantum GX2) was performed after 8 weeks of dietary intervention. The mice were anesthetized with 2.5–3% isoflurane and positioned on the scanning platform. Imaging parameters were set to a current of 100 μA and a voltage of 70 kVp, and the software Analyze 12.0 was employed to quantify fat content and distribution. Moreover, we used the following formula, (body weight − fat volume × fat density)/body weight, to determine the lean mass of mice.

### Determination of eicosapentaenoic acid (EPA)

Peripheral blood (PB) was collected into an EDTA-K2 anticoagulant Vacutainer tube. Plasma was obtained by centrifugation and stored at −80 °C. The levels of EPA were measured using an enzyme-linked immunosorbent assay (ELISA) for mouse samples (Cat.No: MM-45902M1) or human samples (Cat.No: MM-61024H1) according to the manufacturer’s instructions (MEIMIAN, Jiangsu, China). The detection range for mouse samples was 5–400 μmol/L, while that for human samples was 10–240 pg/mL.

### CRISPR-Cas9 knockout

OGT−/−3T3-L1 cell lines were generated by chemical transfection of a Cas9-sgRNA ribonucleoprotein (RNP) complex (RNA-protein complex) synthesized via chemical methods. A total of 5 × 10^5^ cells were seeded into each well of a 24-well plate and transfected with the RNP transfection complex according to the manufacturer’s instructions. Following incubation, cells were harvested, and genomic DNA was extracted for Sanger sequencing to identify the sgRNA with the highest editing efficiency. Selected cells were then single-cell cloned by limiting dilution into 96-well plates. Knockout clones were confirmed by Western blot analysis. The following is the specific information of the sgRNA sequences: sgRNA1-Ogt: Forward primer (F): GCT TGT GTG TAC TAC GAG CA and Reverse primer (R): CAG TAA GCA TCG GGG AAA TG; sgRNA2-Ogt: Forward primer (F): AGC ATT ATC GAC ATG CCT TG and Reverse primer (R): GTA CTG AAG AGC AGA GAC AT; sgRNA3-Ogt: Forward primer (F): AGT GAT GGC CGA TTG CGT GT and Reverse primer (R): TAT GCC TTG AGC CCG GAT GA.

### Statistical analyses

Data in all figures are expressed as mean ± standard error of mean (SEM) and are representative of at least two independent trials. For the data with a normal distribution and homogeneity of variance, Student’s unpaired *t* test was used for the statistical differences between two groups, and one-way analysis of variance (ANOVA) was applied to compared the means of three or more independent groups with Prism 10 software (GraphPad, San Diego, CA). For the non-normally distributed data, the statistical differences between two groups were analyzed using the Mann–Whitney U test, a non-parametric alternative to the T-test. For comparisons involving three groups, the Kruskal–Wallis H test, a non-parametric equivalent of ANOVA, was employed to assess statistical differences. Statistical analysis of gender differences was performed using the Chi-square test (unpaired test). Linear regression was analyzed using IBM SPSS Statistics 21 (SPSS, Chicago, IL, USA). Statistical significance is denoted as *p < 0.05, **p < 0.01, ***p < 0.001, **p < 0.0001.

## Supplementary information

Supplementary figures

Full and uncropped western blots

## Data Availability

All data generated or analyzed during this study are included in this published article [and its supplementary information files].
