# Supplementary material for: Adipocytes regulate monocyte development through the OGT-NEFA-CD36/FABP4 pathway in high-fat diet-induced obesity
Source: Cell Death Dis. 2025 May 19;16(1):401. doi: 10.1038/s41419-025-07721-x (PMC12089399; doi:10.1038/s41419-025-07721-x)
Supplement: Supplementary file 1 — Supplementary figures [file 41419_2025_7721_MOESM1_ESM.docx]

**Supplementary Figures**


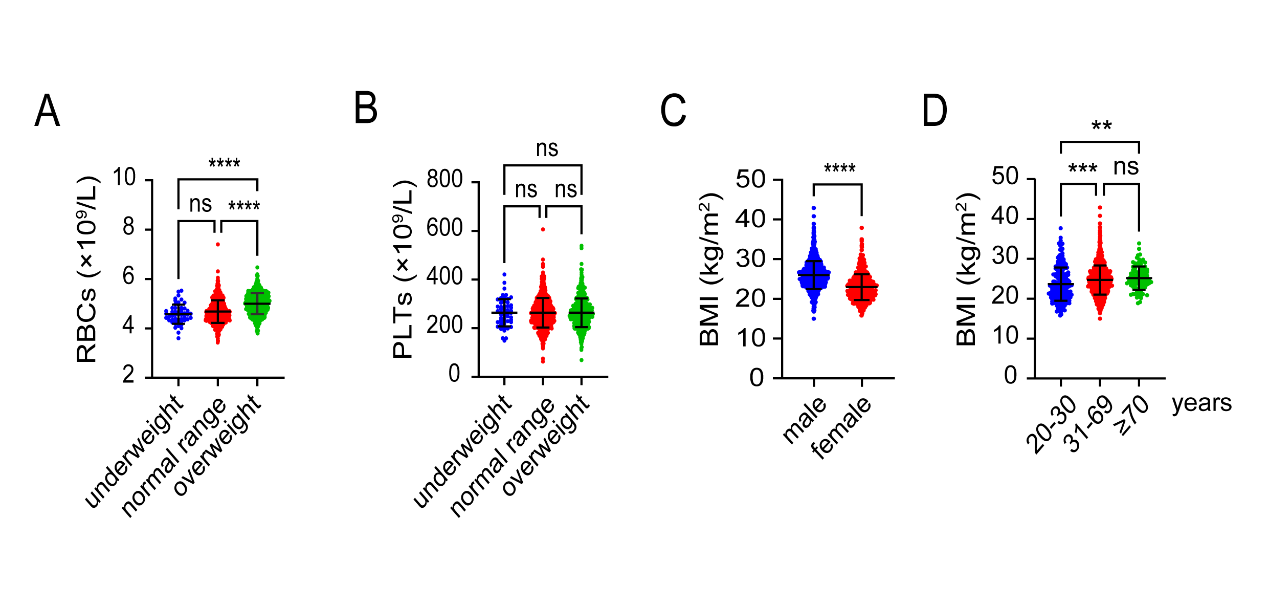


Supplementary Figure 1. Obesity as an independent factor influencing peripheral monocyte levels. (A-B) Absolute values of RBC and PLT in underweight, normal weight, and overweight groups in peripheral blood. (C) The values of BMI in male and female groups. (D) The values of BMI across different age groups. Data are presented as mean ± SEM. *p < 0.05, **p < 0.01, ***p < 0.001, ****p < 0.0001, ns, not significant.


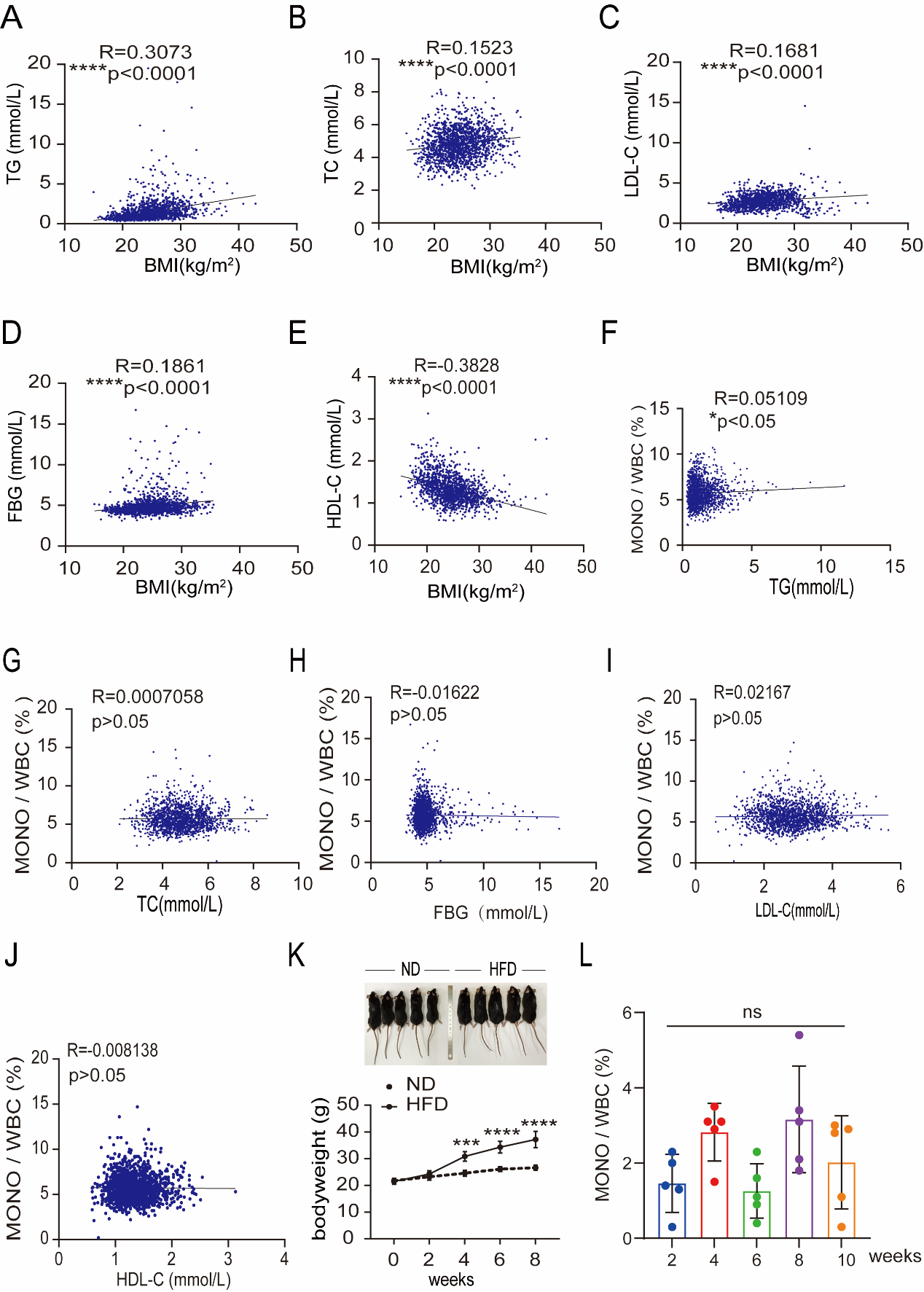


Supplementary Figure 2. HFD-induced obesity is associated with serum dyslipidemia, particularly elevated NEFA levels. (A-E) Correlation analysis of serum levels of TG, TC, HDL-C, LDL-C and FBG with BMI. (F-J) Correlation analysis of serum levels of TG, TC, FBG, LDL-C and HDL-C with proportions of MONO/WBC in peripheral blood. (K) Body weight curves of mice in the HFD-WT (n=7) and HFD-OGT-AKO (n=7) groups throughout the HFD feeding period. (L) The change in the proportion of monocytes in PB of mice in the ND group (n=5) over the course of ND feeding for 10 weeks.


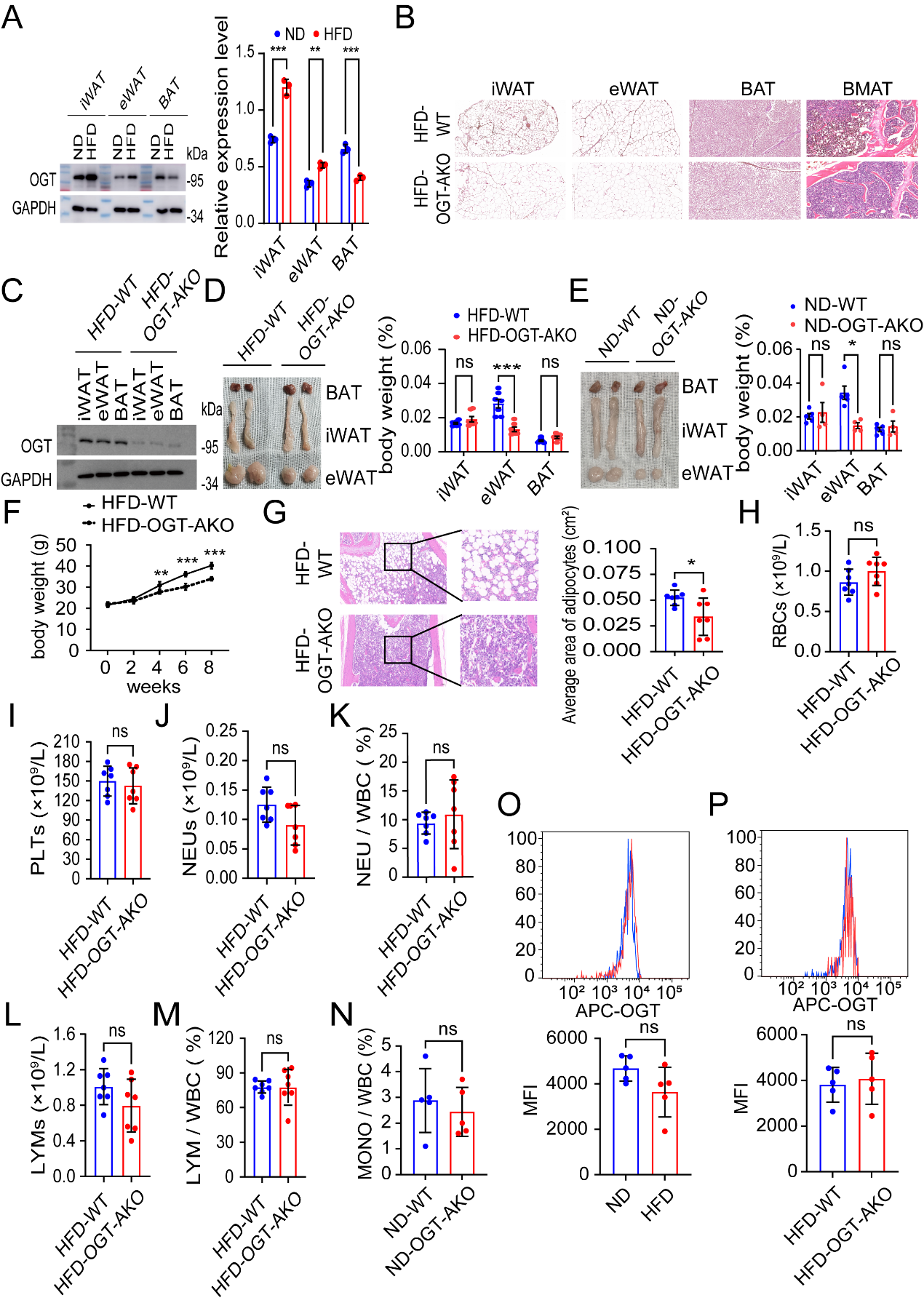


Supplementary Figure 3. Adipocyte OGT contributes to the monocyte increase in HFD-induced obesity. (A) The protein level of OGT in different part of body in adipocytes in ND and HFD groups. Data reported as from n = 3 independent biological replicates. (B) Representative images of OGT expression in iWAT, eWAT, BAT and BMAT in HFD-WT and HFD-OGT-AKO groups. (C) Western blotting analysis in adipose tissue from HFD-WT and HFD-OGT-AKO groups. (D) Representative images of BAT, iWAT, and eWAT from HFD-WT and HFD-OGT-AKO groups. Tissue weights, presented as % of body weight in HFD-WT (n=7) and HFD-OGT-AKO (n=7) groups mice described in (D). (E) Representative images of BAT, iWAT, and eWAT from ND-WT and ND-OGT-AKO groups. Tissue weights, presented as % of body weight in ND-WT (n=5) and ND-OGT-AKO (n=4) groups mice described in (E). (F) Body-weight curves of mice in HFD-WT (n=7) and HFD-OGT-AKO (n=7) groups during HFD feeding time. (G) H&E staining and quantification of BMAds from HFD-WT (n=7) and HFD-OGT-AKO (n=7) groups. And the statistical histograms were shown on the right. (H-I) Statistics of absolute value of RBC and PLT proportion in PB from HFD-WT (n=7) and HFD-OGT-AKO (n=7) groups. (J-K) Statistics of absolute value of NEU and NEU/WBC proportion in PB from HFD-WT (n=7) and HFD-OGT-AKO (n=7) groups. (L-M) Statistics of absolute value of LYM and LYM/WBC proportion in PB from HFD-WT (n=7) and HFD-OGT-AKO (n=7) group. (N) Statistics of MONO/WBC proportion in PB from ND-WT and ND-OGT-AKO groups. (O) MFI of OGT in monocytes (CD11b^+^Ly6C^high^ cells) in ND-WT (n=5) and ND-OGT-AKO (n=5) group. (P) MFI of OGT in monocytes (CD11b^+^Ly6C^high^ cells) in HFD-WT (n=5) and HFD-OGT-AKO (n=5) groups. Data were presented as mean ± s.e.m.^*^p＜0.05，^**^p＜0.01, ^***^p＜0.001, ^****^p＜0.0001, ns, no significance.


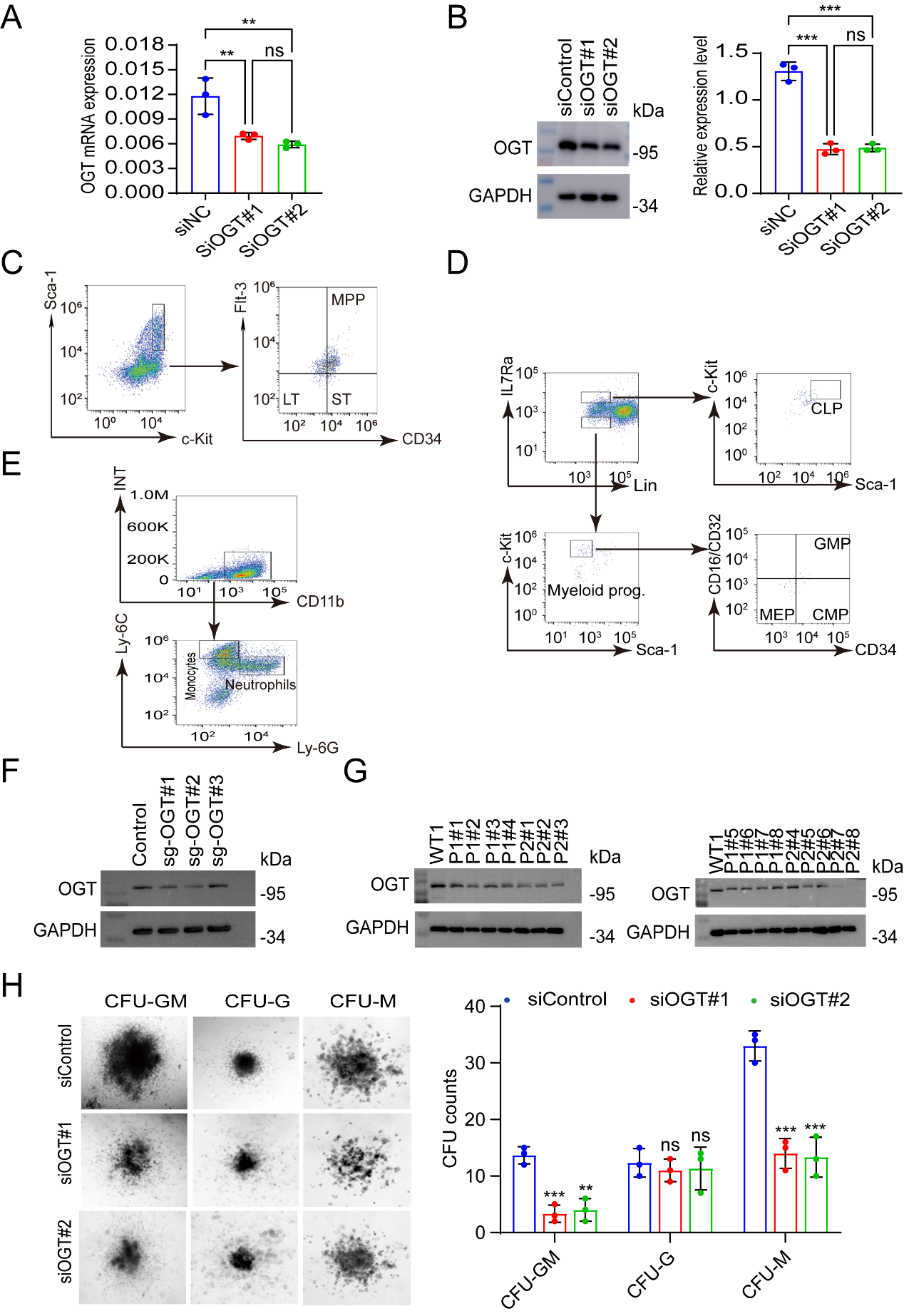


Supplementary Figure 4. Adipocyte OGT regulates the differentiation of hematopoietic stem cells into mature monocytes. (A) The mRNA and protein levels of OGT in adipocytes in siControl and siOGT groups. Data reported as from n = 3 independent biological replicates. (B) Western blot analysis of OGT in adipocytes in siControl and siOGT groups. And the statistical histograms were shown on the right. Data reported as from n = 3 independent biological replicates. (C) The LSKs, LT-LSKs, ST-LSKs, and MPPs proportion analysis of BM in mice was shown. (D) The Myeloid progenitor cells, GMP, MEP, CLP and CMP proportion analysis of BM in mice was shown. (E) The MONO and NEU proportion analysis of BM in mice was shown. (F) Western blot analysis of OGT in adipocytes transfected with three sgRNAs by Crispr-cas9 system. (G) Western blot analysis of OGT in adipocytes in monoclonal cells. (H) Comparison of colony forming units after 14 days of culture in methylcellulose-based medium. And the statistical histograms were shown on the right. Data reported as from n = 3 independent biological replicates. Data were presented as mean ± s.e.m. ^*^p＜0.05，^**^p＜0.01, ^***^p＜0.001, ^****^p＜0.0001, ns, no significance.


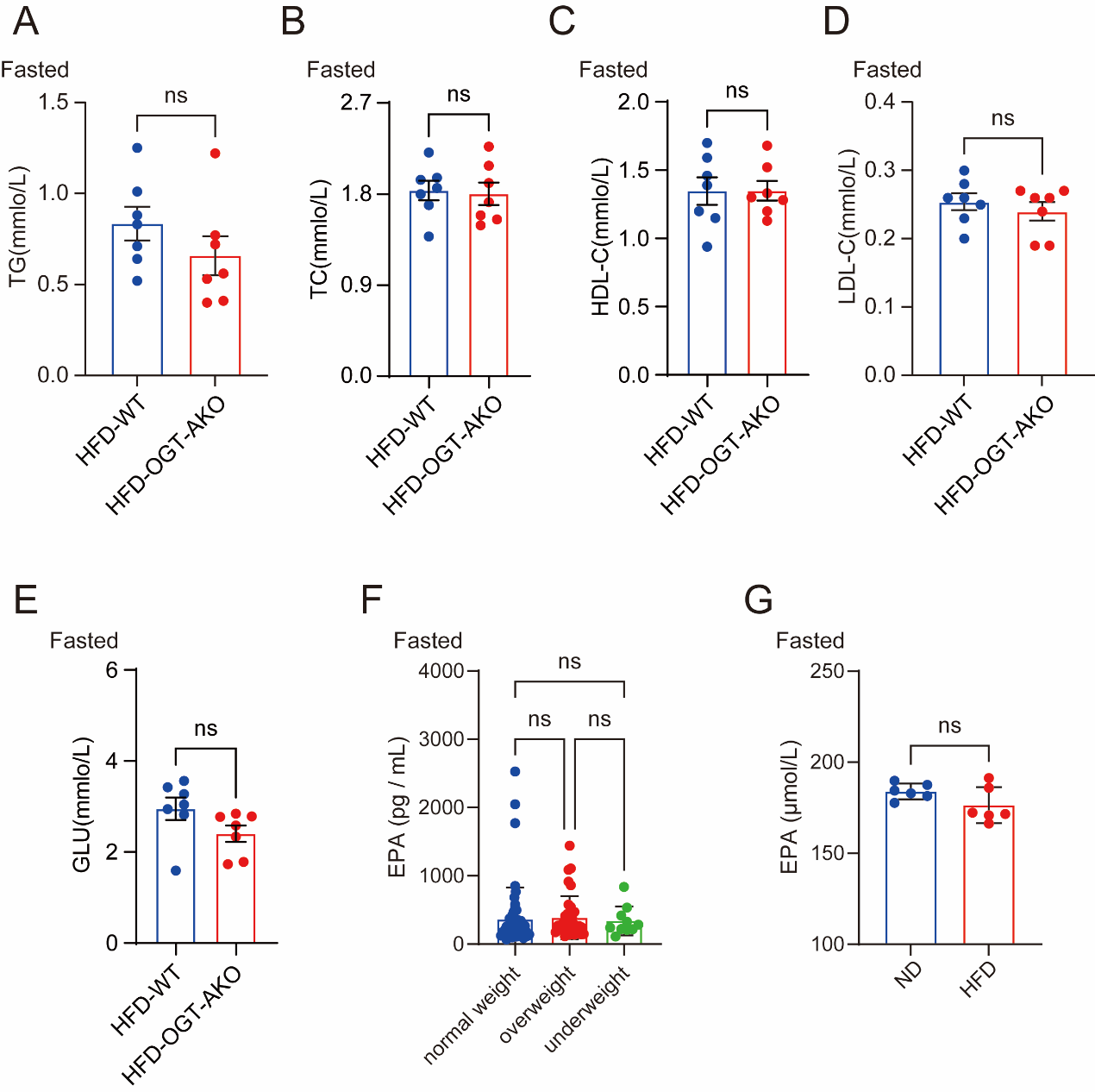


Supplementary Figure 5. Regulatory role of NEFA in adipocyte OGT-mediated monocyte increase in obesity. (A-E) Serum levels of TG, TC, HDL-C, LDL-C, and GLU in HFD-WT (n=7) and HFD-OGT-AKO (n=7) groups in 12h fasted state. (F) Serum levels of EPA in normal weight, overweight and underweight groups in 12h fasted state. (G) Serum levels of EPA in ND (n=6) and HFD (n=6) groups in 12h fasted state. Data were presented as mean ± s.e.m. ^*^p＜0.05，^**^p＜0.01, ^***^p＜0.001, ^****^p＜0.0001, ns, no significance.
