# Supplementary material for: Adipocytes regulate monocyte development through the OGT-NEFA-CD36/FABP4 pathway in high-fat diet-induced obesity
Source: Cell Death Dis. 2025 May 19;16(1):401. doi: 10.1038/s41419-025-07721-x (PMC12089399; doi:10.1038/s41419-025-07721-x)
Supplement: Supplementary file 2 — Full and uncropped western blots [file 41419_2025_7721_MOESM2_ESM.docx]

**Full and uncropped western blots**


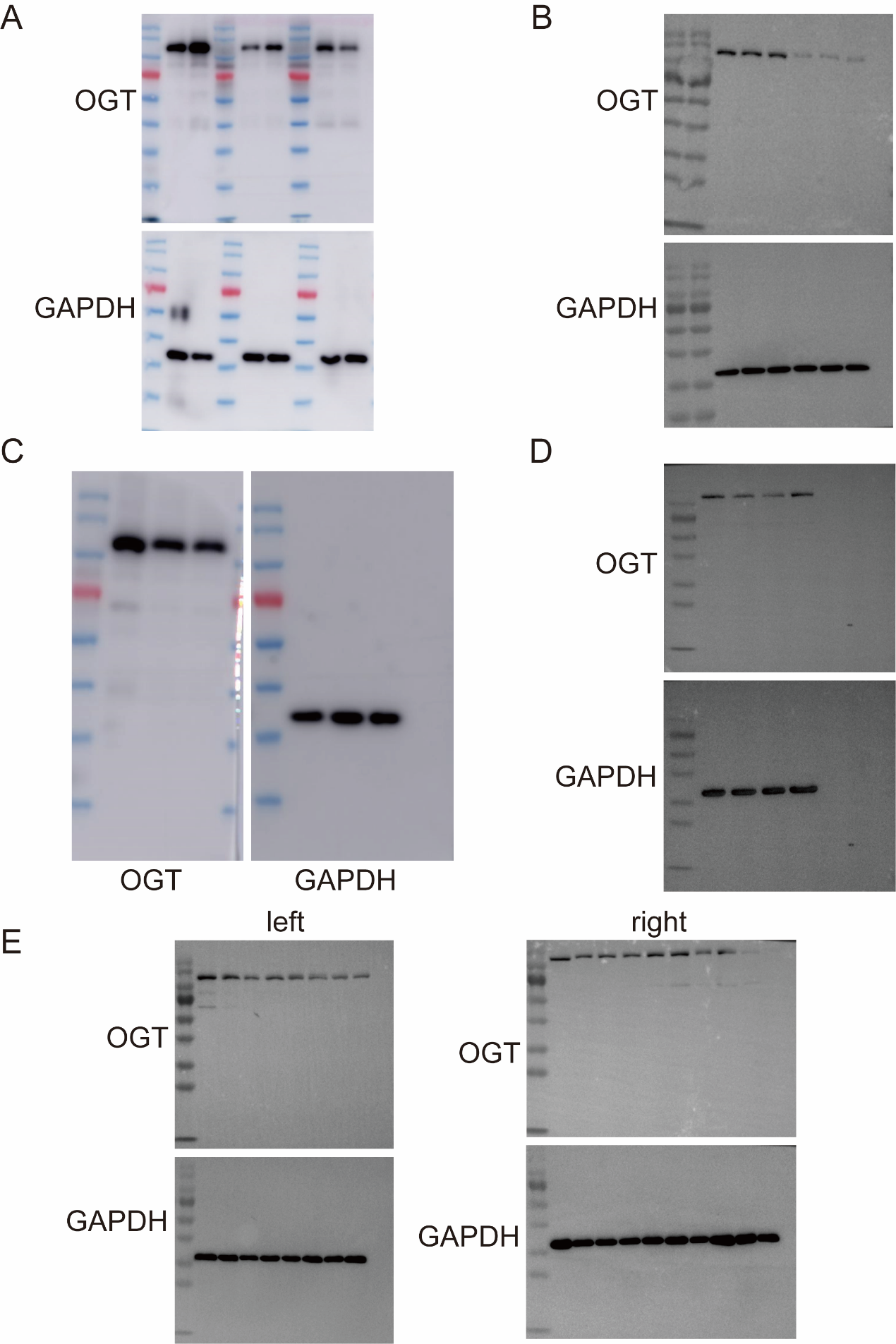


**Supplemental Material. Full and uncropped western blots. (A)** Western blot schematic of supplementary figure 3A. **(B)** Western blot schematic of supplementary figure 3C. **(C)** Western blot schematic of supplementary figure 4B. **(D)** Western blot schematic of supplementary figure 4F. **(E)** Western blot schematic of supplementary figure 4G.
